# LC–ESI–FT–MS^n^ Metabolite Profiling of *Symphytum officinale* L. Roots Leads to Isolation of Comfreyn A, an Unusual Arylnaphthalene Lignan

**DOI:** 10.3390/ijms21134671

**Published:** 2020-06-30

**Authors:** Gilda D’Urso, Milena Masullo, Jacqueline Seigner, Yvonne M. Holper-Schichl, Rainer de Martin, Alberto Plaza, Sonia Piacente

**Affiliations:** 1 Dipartimento di Farmacia, Università degli Studi di Salerno, Via Giovanni Paolo II, 84084 Salerno, Italy; gidurso@unisa.it (G.D.); mmasullo@unisa.it (M.M.); 2 Department of Vascular Biology, Medical University of Vienna, 1090 Vienna, Austria; jacqueline.seigner@meduniwien.ac.at (J.S.); rainer.demartin@meduniwien.ac.at (R.d.M.); 3 The Procter & Gamble Company Wiedner Gürtel 13, 1100 Vienna, Austria; holperschichl.y@pg.com; 4 R&D, P&G Health Germany GmbH, Alsfelder Str. 17, 64289 Darmstadt, Germany

**Keywords:** *Symphytum officinale*, comfrey roots, LC–ESI–Orbitrap–MS, phenylpropanoids, comfreyn A

## Abstract

Preparations of comfrey (*Symphytum officinale* L.) roots are used topically to reduce inflammation. Comfrey anti-inflammatory and analgesic properties have been proven in clinical studies. However, the bioactive compounds associated with these therapeutic activities are yet to be identified. An LC–ESI–Orbitrap–MS^n^ metabolite profile of a hydroalcoholic extract of comfrey root guided the identification of 20 compounds, including a new arylnaphthalene lignan bearing a rare δ-lactone ring, named comfreyn A. Its structure was determined using extensive 2D NMR and ESI–MS experiments. Additionally, the occurrence of malaxinic acid, caffeic acid ethyl ester, along with the lignans ternifoliuslignan D, 3-carboxy-6,7-dihydroxy-1-(3′,4′-dihydroxyphenyl) -naphthalene, globoidnan A and B, and rabdosiin was reported in *S. officinale* for the first time. These results helped to redefine the metabolite profile of this medicinal plant. Finally, caffeic acid ethyl ester and comfreyn A were found to significantly inhibit E-selectin expression in IL-1β stimulated human umbilical vein endothelial cells (HUVEC), with EC values of 64 and 50 µM, respectively.

## 1. Introduction

*Symphytum officinale* L. (Boraginaceae) known as comfrey, is a perennial herbaceous plant commonly found in Europe, Asia, and North America [1,2]. Comfrey is used in folk medicine for the treatment of diarrhea, bronchitis, tuberculosis, ulcers, and hemorrhoids [3]. Nowadays, clinical trials have demonstrated the efficacy and safety of its topical preparations [2,4]. Comfrey roots contain pyrrolizidine alkaloids (PAs) with 1,2 unsaturated necine structures, including lycopsamine, intermedine, their acetyl, and N-oxide derivatives, together with the diester symphytine [2,5,6]. Therefore, care is taken to deplete the PA presence through extraction, and strict limits must be met for the placement on the market of PA containing herbal medicines [7]. PA-depleted extracts are used in over-the-counter topical medicines to reduce inflammation and for the treatment of broken bones, tendon damages, painful joints, and muscles [3]. Indeed, a liquid hydroalcoholic extract of comfrey root was clinically proven to be effective for the treatment of acute upper and lower back pain, gonarthrosis, and blunt injuries [8,9,10].

It was reported that the main constituents of comfrey root include allantoin, polyphenols such as rosmarinic acid, lithospermic acid, ellagic acid, caffeic acid, and abundant mucilage and polysaccharides composed of fructose and glucose units [1,2,3,11]. Moreover, comfrey roots have yielded a number of oleanolic and bidesmosidic triterpene saponins [12,13]. Recently, a phenolic profile of a hydroalcoholic extract from the roots of *Symphytum officinale* L. was reported to contain salvianolic acid isomers [2,14].

At present, medicinal products from comfrey root commercialized on the European market contain only extracts from pyrrolizidine-depleted plant material or are obtained from special cultivars that do not synthesize pyrrolizidine alkaloids [2].

As part of our ongoing work that aims at better understanding the chemical composition of comfrey roots and obtaining preliminary insights on the contribution of its specialized metabolites to the anti-inflammatory activity, we performed an LC–MS^n^ guided fractionation of a mucilage- and PA-depleted extract of comfrey root that showed inhibition of interleukin-1β-induced expression of E-selectin in HUVEC cells, at a concentration of 20 µg/mL [4]. Through these means, 20 compounds were identified, including a new arylnaphthalene lignan, named comfreyn A. Its structure was fully characterized by extensive NMR and ESI–MS analysis.

Moreover, malaxinic acid, caffeic acid ethyl ester, along with the lignans ternifoliuslignan D, 3-carboxy-6,7-dihydroxy-1-(3′,4′-dihydroxyphenyl)-naphthalene, globoidnan A and B, and rabdosiin were reported for the first time in *S. officinale*.

## 2. Results and Discussion

**Figure 1:**
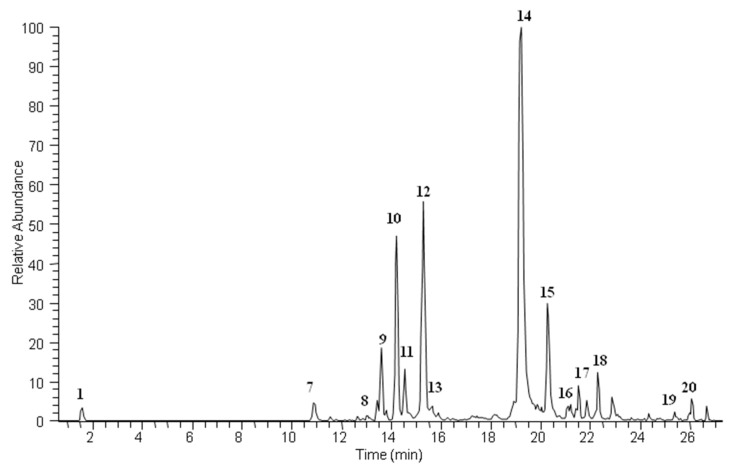
LC–MS profile in negative ion mode of *Symphytum officinale* ethanol extract.

**Figure 2:**
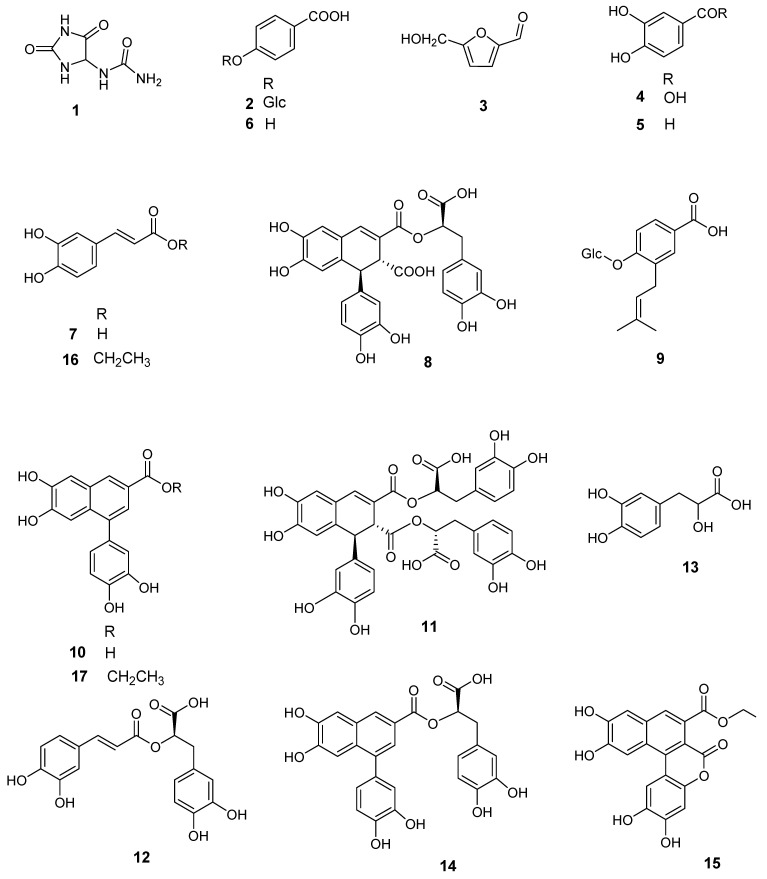
Compounds isolated from the roots of *Symphytum officinale*.

A hydroalcoholic liquid extract of *S. officinale* root was analyzed by LC–ESI/HR/MS, using the “data dependent scan” mode in which precursor ions corresponding to the most intense peaks were selected. A preliminary metabolite profiling showed 20 main compounds corresponding to different structural classes (Figure 1 and Figure 2).

Some of these metabolites were identified by a comparison of their accurate masses, characteristic fragmentation patterns, and retention times to those of authentic standards. On the basis of the above approach, the occurrence of allantoin (**1**), protocatechuic acid (**4**), protocatechuic aldehyde (**5**), p-hydroxy benzoic acid (**6**), caffeic acid (**7**) and its ethyl ester derivative (**16**), and rosmarinic acid (**12**), along with the fatty acids linolenic (**18**), linoleic (**19**), and hydroxypalmitic (**20**) was reported. The remaining compounds could not be unambiguously assigned by HR–ESI–MS, either because their accurate masses did not match any of the compounds reported from *S. officinale* in the literature or because their preliminary NMR data did not support the compounds reported in *S. officinale*, with the same molecular weights. In particular, compound **15** showed an [M-H]^−^ ion at *m/z* 381.0601 in the LC–MS profile, suggesting a molecular formula of C_20_H_14_O_8_, which did not correspond to any molecule reported in the scientific literature. To fully characterize compounds **2**–**3**, **8**–**11**, and **13**–**17**, we first removed the mucilage fraction of the extract by partitioning the liquid extract with ethyl acetate. Then, the organic phase was subjected to size exclusion chromatography on a Sephadex LH-20 column. Subsequently, fractions containing the above-mentioned compounds were purified by reverse-phase HPLC. Their structures were fully characterized by extensive 1D- and 2D-NMR experiments, as well as MS/MS analysis (see Table 1).

Our NMR analysis confirmed **2** and **3** as p-hydroxy benzoic acid glucoside [15] and 5-hydroxymethyl-2-furfural [16], respectively. However, it is worth mentioning that **3** could be an artefact of the extract preparation due to the use of a cation-exchange resin to deplete the PAs [17]. Indeed, the resin might cause degradation of sugar residues (e.g., fructose, glucose, sucrose) to form 5-hydroxymethyl-2-furfural [16]. Compound **9** showed a major ion peak at *m/z* 367.1384 [M-H]^−^, supporting the molecular formula C_18_H_24_O_8_. Its MS/MS spectrum showed a fragment ion at *m/z* 205.09 [M-H-162]^−^ suggesting the presence of a hexose unit. Its structure was established through NMR as malaxinic acid [18]. Interestingly, this was the first report of this glucosylated and prenylated phenolic acid (**9**), in *S. officinale*.

The HR–ESI–MS spectra of **8**, **11**, and **14** supported the molecular formulae of C_27_H_22_O_12_, C_36_H_30_O_16_, and C_26_H_20_O_10_, respectively. Analysis of their NMR and MS/MS data allowed us to identify **14** and **8** as globoidnan A [19] and B [20], respectively, whereas compound **11** was established as (+)-rabdosiin in agreement with its optical rotation [21] (see Table S1). To our knowledge, this is the first report of these lignans in roots of *S. officinale*. Interestingly, the molecular formulae and fragmentation patterns of **8**, **11**, and **14** perfectly matched those of lithospermic acid, salvianolic acid B, and salvianolic acid C, respectively. These lithospermic acid isomers were previously identified in roots of *S. officinale* by LC–MS or UV and IR spectroscopy, however, their structures were not confirmed by NMR experiments [2,22]. The molecular formula of compound **13** was established as C_9_H_10_O_5_ and the structural elucidation afforded by the NMR analysis, allowed the identification of compound **13** as α-hydroxyhydrocaffeic acid [23].

MS/MS fragmentation patterns suggested that **10** and **17** bear a similar structural framework as that of globoidnan A (**14**). Indeed, interpretation of the NMR data confirmed this hypothesis establishing the structure of **10** as 3-carboxy-6,7-dihydroxy-1-(3′,4′-dihydroxyphenyl)-naphthalene and **17** as its ethyl ester derivative ternifoliuslignan D [24].

The HR–ESI–MS spectrum of compound **15** (*m/z* 381.0601 [M-H]^−^, calculated for C_20_H_13_O_8_, 381.0610) supported a molecular formula of C_20_H_14_O_8_. The ESI/MS/MS spectra of **15** displayed an intense ion at *m/z* 353.02 ([M-H-28]^−^), suggesting the presence of an ethyl group. The MS^3^ spectra obtained from this product ion displayed fragment ions at *m/z* 309.10 ([M-H-44]^−^) and 265.10 ([M-H-44-44]^−^), corresponding to the loss of two CO_2_ molecules. The analysis of the ^13^C and HSQC NMR data for **15** (Table 2) suggested a highly unsaturated molecule bearing 16 aromatic carbons, whereas its ^1^H NMR spectrum displayed five aromatic singlets at δ 8.30 (H-8), 8.02 (H-6′), 7.67 (H-4), 7.34 (H-5), 6.91 (H-3′). Additionally, ^1^H NMR of **15** showed characteristic signals of an ethoxy group at δ 4.43 (2H, q, *J* = 7.0 Hz) and 1.41 (3H, t, *J* = 7.0 Hz). Moreover, a detailed analysis of the HMBC spectrum confirmed the presence of two main fragments. In particular, key long-range correlations between the aromatic proton H-4 and the carbon resonances at δ 172.2 (C-9), 130.5 (C-3), 113.4 (C-2), 133.7 (C-4a), 112.4 (C-5), and 124.5 (C-8a), H-5 and the carbon resonances at δ 133.7, 151.3 (C-6), 150.3 (C-7), and 124.5, and H-8, and the carbon resonances at δ 136.1 (C-1), 151.3, and 133.7, established the occurrence of a 1,2-disubstituted 6,7-dihydroxy-naphthalene-3-carboxylic acid. The structure of the second fragment was established as 1-substituted benzene-2,4,5-triol based on HMBC correlations from protons H-3′ and H-6′ to the carbon resonances at δ 111.5 (C-1′), 143.8 (C-2′), 149.6 (C-4′), and 147.1 (C-5′). In turn, connectivity between these two fragments was deduced from the HMBC correlation from H-6′ to C-1. In addition, an HMBC correlation from the equivalent methylene at δ 4.43 (H-1′’) to the carbonyl at δ 172.2 linked the ethoxy residue to the naphthalene moiety. Inspection of this partial structure revealed that it contained 13 of the required 14 degrees of unsaturation and only one of the two carbonyls was assigned. Consequently, the phenol ring and the 6,7-dihydroxy-naphthalene-3-carboxylic acid residue had to connect via formation of a lactone ring to satisfy the unsaturation index and molecular formula. HMBC experiments with long-range delays ranging from 2.75 to 10 Hz were performed. The HMBC spectrum, optimized for the ^n^*J*(C,H) coupling of 5 Hz, corresponding to a delay time Δ2 = 100 ms, showed four-bonds of long-range correlations and stronger two-bond long-range correlations. Finally, the structure of **15** was assembled as follows.

**Figure 3:**
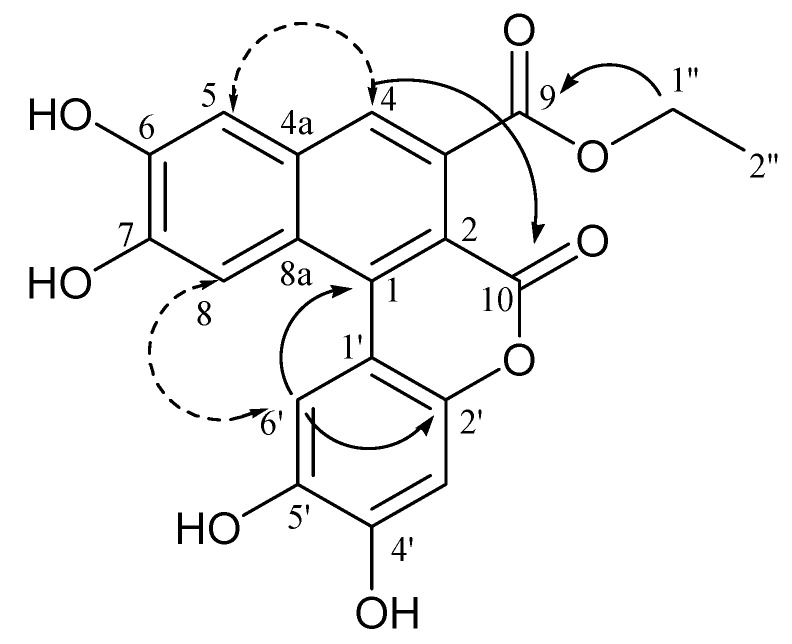
Key HMBC (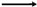) and ROESY (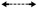) correlations of compound **15**.

A ^4^*J*_CH_ HMBC correlation from H-4 to the carbon resonance at δ 162.5 (C-10) allowed the deduction of the linkage of the carbonyl group to C-2, whereas an ROE correlation between H-6′ and H-8, along with a ^4^*J*_CH_ HMBC correlation from H-6′ to C-2 indicated the esterification site at C-2 (Figure 3). Therefore, comfreyn A (**15**) was established as an arylnaphthalene lignan bearing an unusual δ-lactone ring, corresponding to a coumarin skeleton.

It is worth mentioning that the natural occurrence of the ethyl esters **15**–**17** was ascertained by HPLC-UV analysis of the methanol extract of dried roots, to prove their presence in comfrey not as artefacts due to the use of ethanol but as extraction solvent (Figure S1). Figures S2–S18 (NMR spectra of compounds **8**, **11**, **14** and **15**) and Table S1 (^13^C and ^1^H NMR data of compounds **8**, **11** and **14**) are reported in the Supplementary Materials.

**Figure 4:**
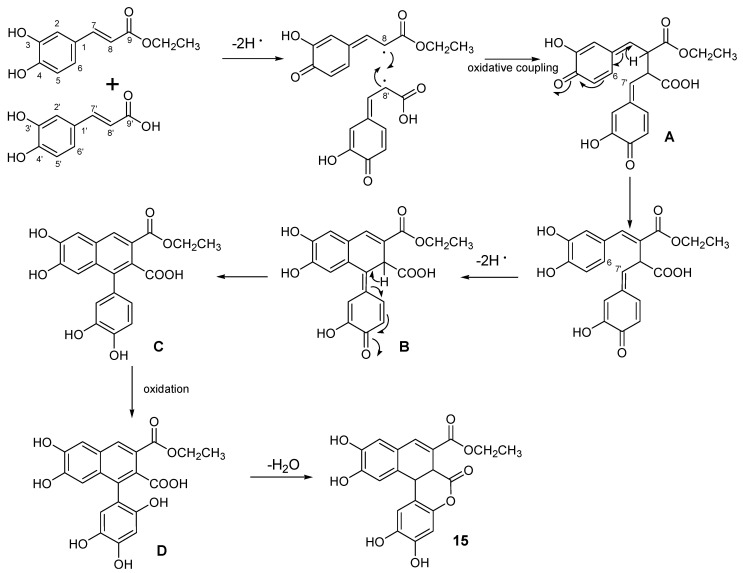
Proposed biosynthetic pathway for compound **15**.

From a biosynthetic point of view, this work highlighted comfrey roots as a great source of phenylpropanoids, including monomeric phenylpropanoids (**7**, **13**, and **16**), dimeric phenylpropanoids with the two C_6_C_3_ moieties linked via an esther function (**12**) and lignans (**8**, **10**, **11**, **14**, **15**, and **17**). The latter are a class of secondary metabolites derived from two phenylpropanoid units, linked by a C–C bond between C8 and C8ʹ, which could show a remarkable structural diversity [25]. Indeed, the putative biosynthetic pathway of comfreyn A probably originated from caffeic acid and its ethyl ester analogue (Figure 4).

As previously reported by Daquino et al., removal of a hydrogen atom from both the p-phenolic group of caffeic acid and its ethyl ester analogue originated phenoxy radicals with the unpaired electron at the β position, which might couple to each other (8–8′coupling), generating a reactive bis-quinonemethide (A) [25]. Its tautomer underwent an oxidative intramolecular cyclization (6–7′) (B). Then, the subsequent oxidative step introducing a phenolic function on the tautomer C produced the intermediate D. Finally, the structure of **15** was completed by a condensation step that led to the formation of a lactone ring.

It was reported that interleukin-1β (IL-1β) induced E-selectin mRNA levels were inhibited by comfrey extract by approximately 70% at the starting concentration of 20 µg/mL [4]. E-selectin is a NF-κB-dependent target gene and IL-1β is a well-described pro-inflammatory mediator that exerts an important role in the regulation of immune and inflammatory responses, in particular those involving sterile insults, such as trauma and blunt injuries. Thus, the anti-inflammatory activity of compounds **1**, **5**, **9**, **12**, **14**, **15**, and **16** was tested using IL-1-stimulated human umbilical vein endothelial cells (HUVEC) and analyzed by real time PCR, determining the fold change in mRNA expression of E-selectin. The results are shown in Table 3. Caffeic acid ethyl ester (**16**) was the most active compound inhibiting E-selectin expression by 79.6 ± 4%, at a concentration of 64 µM, followed by comfreyn A (**15**), which inhibited E-selectin expression by 51.5 ± 5.3% at 50 µM. Globoidnan A (**14**), protocatechuic aldehyde (**5**), and malaxinic acid (**9**) were significantly inhibited E-selectin expression inhibited but with less potency, while allantoin (**1**) and rosmarinic acid (**12**) did not show any activity at concentrations as high as 250 µM.

Surprisingly, the anti-inflammatory activity of the tested compounds did not support the activity displayed by the mucilage- and PA-depleted extract of comfrey root at 20 µg/mL, in our in vitro anti-inflammatory model [4]. These results might suggest that the activity of the extract could be due to a combined or synergistic effects of the compounds herein occurring in the extract, belonging to the phenolic class [26]. Indeed, our phytochemical investigation allowed us to determine the chemical composition of the comfrey root, highlighting the presence of natural compounds previously reported to show anti-inflammatory activity, which could partially explain the biological activity shown by the extract. Among these compounds, caffeic acid ethyl ester (**16**) was reported to suppress in vitro or in mouse skin NF-κB activation and its downstream inflammatory mediators, iNOS, COX-2, and PGE2 [27], and ternifoliuslignan D (**17**) was shown to inhibit NO, TNF-α, and PGE2 production and also to suppress the expression of iNOS, NF-κB/p65, and COX-2 in LPS-induced RAW 264.7 cells [24]. Additionally, human peripheral blood mononuclear cells (PBMCs) stimulated by lipopolysaccharide (LPS), rabdosiin (**11**), and rosmarinic acid (**12**) significantly decreased secretion of TNF-α, IL-1b, IL-2, and IL-6, at a concentration of 100 µM. Globoidnan A (**14**) significantly reduced the production of only TNF- α [28]. 

## 3. Materials and Methods

### 3.1. General Procedures

Optical rotation of compound **11** was measured on an Autopol IV (Rudolph Research Analytical, Hackettstown, NJ, USA) polarimeter. IR measurements were carried out on a Bruker IFS-48 spectrometer. UV spectra were obtained on a Beckman DU 670 spectrometer. NMR experiments were acquired in methanol-*d*_4_ (99.95%, Sigma-Aldrich, Milan, Italy) on a Bruker DRX-600 spectrometer (Bruker BioSpin GmBH, Rheinstetten, Germany), equipped with a Bruker 5 mm TCI CryoProbe at 300 K. Data processing was carried out with Topspin 3.2 software (Bruker BioSpin, Rheinstetten, Germany). The ROESY spectra were acquired with t_mix_ = 400 ms. Size exclusion chromatography was performed using Sephadex LH-20 (GE Healthcare, Sigma Aldrich, Milan, Italy).

### 3.2. Reagents and Solvents

Ethanol and methanol for extraction were purchased from VWR international PBI S.r.l. (Milan, Italy). Water and Acetonitrile for HPLC were purchased from VWR international PBI S.r.l. (Milan, Italy). Water, acetonitrile, and formic acid (all of LC–MS grade) were purchased from Merck (Darmastadt, Germany). Allantoin standard was purchased from Sigma Aldrich (Milan, Italy).

### 3.3. Sample Extraction

Comfrey roots were harvested in Serbia (region Vojvodina) in 2016. The botanical identification of the roots was done in accordance with the *Symphytum officinale* monograph of the German homeopathic pharmacopeia. The comfrey roots were extracted with ethanol 60% (*v/v*) and PAs were removed with a cation exchange resin, and a PA-depleted hydroalcoholic (20% EtOH *w/w*) liquid extract (DER 1:2) was obtained. The extract was supplied by Procter Health Care International & P&G Health Austria GmbH & Co. OG. To remove the mucilage, the ethanol content of 500 g of liquid extract was evaporated and the aqueous phase was partitioned with ethylacetate (1:1), obtaining 50 g of ethyl acetate extract. A total of 3 g of ethyl acetate extract were fractionated on a Sephadex LH-20 (GE Healthcare, Sigma Aldrich, Milan, Italy) column (100 × 5 cm), using methanol as the mobile phase, affording 80 fractions (8 mL), monitored by LC-ESI-HR-MS (Thermo Fisher Scientific, Bremen, Germany) analysis.

### 3.4. LC–ESI/LTQOrbitrap/MS Analysis

The secondary metabolite profile of the hydroalcoholic extract of comfrey roots was obtained using an HPLC method, coupled with a hybrid mass spectrometer, which combined the linear trap quadrupole (LTQ) and Orbitrap mass analyzer. All experiments were carried out using a Thermo scientific liquid chromatography system constituted of a quaternary Accela 600 pump and an Accela autosampler, connected to a linear Trap-Orbitap hybrid mass spectrometer (LTQ-Orbitrap XL, Thermo Fisher Scientific, Bremen, Germany), with electrospray ionization (ESI). Separation was performed on a Kinetex EVO C18 5 µm (150 mm × 2.10 mm) column (Phenomenex Aschaffenburg, Germany), using a flow rate of 0.2 mL/min and a mobile phase consisting of a combination of A (0.1% formic acid in water, *v/v*) and B (0.1% formic acid in acetonitrile, *v/v*). A linear gradient program starting with isocratic at 5% B in 5 min, then from 5 to 23% B in 5 min, held at 23% B for 5 min, from 23 to 55% B in 7 min, from 55 to 95% B in 5 min, held at 95% B for 5 min was used. The mass spectrometer operated in negative ion mode. The ESI source parameters were as follows—capillary voltage –48V; tube lens voltage –176.47; capillary temperature 280 °C; sheath and auxiliary gas flow (N_2_), 15 (arbitrary units) and 5 (arbitrary units), and sweep gas 0 spray voltage 5 kV. The mass range was from 120 to 1600 *m/z* with a resolution of 30,000. For the fragmentation study, a data dependent scan was performed, selecting precursor ions corresponding to the most intense peaks in the LC–MS analysis. Xcalibur software version 2.1 was used for instrument control, data acquisition, and data analysis.

### 3.5. Isolation Procedure

On the basis of the results obtained from the LC–ESI–HR–MS analyses, Sephadex fractions were further purified by semipreparative HPLC–UV. Indeed, the obtained dried fractions were diluted in acetonitrile (at a concentration of 10 mg dry residue per 100 µL), and submitted to semipreparative HPLC–UV separations, using a Phenomenex C18 Synergi-Hydro-RP (250 mm × 10 mm, 10 μm) column on an Agilent 1260 Infinity system (Agilent Technologies, Palo Alto, CA, USA), equipped with a binary pump (G-1312C), and a UV detector (G-1314B). The mobile phase consisted of solvent A (H_2_O + 0.1 % formic acid) and solvent B (CH_3_CN + 0.1% formic acid). The analyses were performed at room temperature without thermostating. The peaks collected at HPLC–UV were evaporated to dryness in vacuum and were freeze-dried before NMR analysis. Fraction 23–26 (10 mg) corresponded to compound **12**; fraction 40–42 (10 mg) corresponded to compound **14**. Fractions 19–22 (51.6 mg) were purified using a linear gradient program at a flow rate of 2 mL/min at a wavelength of 254 nm (from 0 to 20% B in 15 min; from 20 to 40% B in 10 min; from 40 to 60% B in 10 min; from 60 to 100% B in 11 min, and then followed by 6 min at 100% B), to afford compounds **2** (1.2 mg, Rt = 10.3 min), **3** (1.3 mg, Rt = 13.5 min), **6** (1.5 mg, Rt = 11.2 min), and **9** (2.2 mg, Rt = 27.3). Fractions 27–39 (42.3 mg), 43–54 (7.1 mg), and 55–80 (22.2 mg) were purified in the same elution conditions at a flow rate of 2 mL/min and at a wavelength of 280 nm: (0–5 min held at 5% B, from 5% to 20% B in 15 min; from 20% to 50% B in 20 min, from 50% to 100% B in 10 min, and held at 100% B for 5 min). In these conditions, compounds **4** (1.1 mg, Rt = 22.60 min), **5** (1.2 mg, Rt = 27.9min), **7** (1.5 mg, Rt = 29.4min), **8** (1.3 mg, Rt = 31.7 min), **11** (1.6 mg, Rt = 34.3), **13** (1.7 mg, Rt = 35.1 min), and **10** (4.4 mg, Rt = 35.3) were isolated from fractions 27–39; compound **15** (1.6 mg Rt = 41.10) was isolated from fractions 43–54, and finally compounds **16** (1.2 mg, Rt = 42.2), and **17** (1.0 mg, Rt = 43.3) were isolated from fractions 55–80. Moreover, 3 g of raw material were extracted with 50 mL of methanol to obtain the methanol extract.

### 3.6. Spectroscopic Data of Compound ***15***

Amorphous yellow solid; C_20_H_14_O_8_; IR *ν*^KBr^_max_ cm^−1^: 3420, 1660, 1615, 1600; ^1^H and ^13^C NMR (methanol-*d*_4_, 600 MHz and 150 MHz) data, see Table 2; ESI-HR-MS *m/z* 381.0601 [M-H]^−^ (calcd. for C_20_H_13_O_8_, 381.0610).

### 3.7. Cell Culture

Primary human umbilical vein endothelial cells (HUVEC) were isolated from umbilical cords, as described previously [29] and maintained in M199 medium (Lonza, Basel, Switzerland) supplemented with 20% FCS (Sigma), 2 mM L-glutamine (Sigma), penicillin (100 units/mL), streptomycin (100 mg/mL), 5 units/mL heparin, and 25 mg/mL ECGS (Promocell, Heidelberg, Germany), and were used up to 5 passages. HUVEC cells were pre-incubated with various concentrations of purified compounds (**1**, **5**, **9**, **12**, **14**, **15**, and **16**) for 30 min, following stimulation with 5 ng/mL IL-1β (R&D Systems) for 90 min. Cells were then analyzed for the change in mRNA expression of E-Selectin (SELE).

### 3.8. Real-Time PCR

Total RNA was isolated using the PeqGold Total RNA Isolation Kit (VWR International, Vienna, Austria), according to the manufacturer’s instructions. A total of 1 µg RNA was reverse transcribed using random hexamers (Fermentas) and murine leukemia virus reverse transcriptase (Thermo Scientific Fisher, Vienna, Austria). Primers were designed using the software “Primer3”. Following primer sequences were used for qPCR: glyceraldehyde 3-phosphate dehydrogenase (GAPDH) forward, 5′-AGAAGGCTGGGGCTCATTT-3′and reverse, 5′-CTAAGCAGTTGGTGGTGCAG-3′; E-Selectin (SELE) forward, 5′-CCTGTGAAGCTCCCACTGA-3′, and reverse 5′- GGCTTTTGGTAGCTTCCATCT-3′. Real-time PCR was done with the SsoAdvanced Universal SYBR Green Supermix (BioRad), using the StepOnePlus instrument (Applied Biosystems, Foster City, CA, USA), and relative mRNA expression was normalized to GAPDH. Fold changes in the mRNA expression were calculated according to the 2-ΔΔCT method. Observed effective concentration and the corresponding inhibition of three independent experiments are given as the percentage of maximum inhibition of the E-Selectin expression, compared to the untreated IL-1β control (Table 3).

## 4. Conclusions

The present study provided a detailed information about the chemical composition of a hydroalcoholic extract of comfrey roots. A new arylnaphthalene lignan, named comfreyn A was isolated and characterized by the extensive use of 1D and 2D-NMR in combination with mass spectrometry. Moreover, this was the first occurrence of malaxinic acid, caffeic acid ethyl ester, along with the lignans ternifoliuslignan D, 3-carboxy-6,7-dihydroxy-1-(3′,4′-dihydroxyphenyl)- naphthalene, globoidnan A and B, and rabdosiin in *S. officinale*. These results, together with the fact that lithospermic acid and the salvianolic acid were not detected, might contribute to redefine the metabolite profile of this medicinal plant. In addition, the anti-inflammatory activity of compounds **1**, **5**, **9**, **12**, and **14**–**16** was tested using IL-1-stimulated human umbilical vein endothelial cells (HUVEC) and were analyzed by real-time PCR, determining the fold change in the mRNA expression of E-selectin. The obtained results showed a weak activity for each pure compound but a good anti-inflammatory activity for the extract. This interesting activity might be due to combined or synergistic effects of the compounds herein occurring in the extract belonging to the phenolic class. These results extend and reinforce the use of *Symphytum* PA-depleted extracts in the preparation of pharmaceutical formulations.

## Figures and Tables

**Table 1:** Secondary metabolites identified by LC–ESI–HR–MS and LC–ESI–HR–MS/MS.

N°	R_t_	[M-H]^−^	[M+H]^+^	Molecular Formula	Δ ppm	MS/MS	Identity
1	1.54	157.0362		C_4_H_6_O_3_N_4_	3.8	140.01/114.03/97.00	allantoin
2	2.42	299.0764		C_13_H_16_O_8_	0.9	137.02	*p*-hydroxybenzoic acid glucoside
3	3.17	-	127.0388	C_6_H_6_O_3_	1	-	5-hydroxymethyl-2-furfural
4	5.25	153.0196		C_7_H_6_O_4_	0.5	-	protocatechuic acid
5	6.79	137.0247		C_7_H_6_O_3_	0.8	-	protocatechuic aldehyde
6	8.07	137.0245		C_7_H_6_O_3_	0.8	93.03	*p*-hydroxybenzoic acid
7	10.88	179.0343		C_9_H_8_O_4_	2.3	-	caffeic acid
8	13.41	537.1033		C_27_H_22_O_12_	1.08	339.05/493.10	globoidnan B
9	13.60	367.1384		C_18_H_24_O_8_	2.6	205.09	malaxinic acid
10	14.21	311.0547		C_17_H_12_O_6_	−0.85	267.06/108.90	3-carboxy-6,7-dihydroxy-1-(3′,4′-dihydroxyphenyl)-naphthalene
11	15.06	717.1449		C_36_H_30_O_16_	−0.15	519.09/475.10/339.05	(+)-rabdosiin
12	15.28	359.0764		C_18_H_16_O_8_	0.6	161.02	rosmarinic acid
13	15.65	197.0448		C_9_H_10_O_5_	1.7	179.03	α-hydroxyhydrocaffeic acid
14	19.21	491.0974		C_26_H_20_O_10_	0.2	311.05/267.06/197.85	globoidnan A
15	20.27	381.0601		C_20_H_14_O_8_	−1.06	353.02/309.10/265.10	comfreyn A
16	21.18	207.0654		C_11_H_12_O_4_	0.1	179.03/135.04/161.02	caffeic acid ethyl ester
17	22.31	339.0863		C_19_H_16_O_6_	0.05	311.05/229.01	ternifoliuslignan D
18	25.56	277.2159		C_18_H_30_O_2_	−1.28	233.22	linolenic acid
19	26.08	279.2315		C_18_H_32_O_2_	−1.14	261.22	linoleic acid
20	28.50	271.2263		C_16_H_32_O_3_	−1.5	225.22	hydroxy-palmitic acid

**Table 2:** ^13^C (150 MHz) and ^1^H NMR (600 MHz) data of compound **15**.

	δ_C_	δ_H_ (*J* in Hz)	HMBC (H→C) Correlations
1	136.2	-	
2	113.8	-	
3	130.5	-	
4	126.6	7.67, s	C-1, C-2, C-4, C-4a, C-8a, C-9, C-10
4a	133.6	-	
5	112.4	7.34, s	C-4, C-8a, C-6, C-7
6	151.3	-	
7	150.2	-	
8	111.5	8.30, s	C-1, C-4a, C-6, C-7
8a	124.5	-	
9	172.1		
10	162.4	-	
1′	111.5	-	
2′	144.0	-	
3′	104.5	6.91, s	C-1, C-1′, C-2′, C-4′, C-5′
4′	149.6	-	
5′	147.1	-	
6′	113.8	8.02, s	C-1, C-1, C-2′, C-4′, C-5′
1″	62.8	4.43, q (7.0)	C-9, C-2″
2″	14.2	1.41, t (7.0)	C-1″

**Table 3:** Inhibitory effect of compounds **1**, **5**, **9**, **12**, **14**, **15**, and **16** on IL-1β induced E-selectin expression.

Compound	EC [µM]	Max. Inhibition (%)
**1**	>250	ND
**5**	120	59.1 ± 16.7
**9**	108	56.2 ± 12.1
**12**	>250	ND
**14**	40	35.1 ± 10.1
**15**	50	51.5 ± 5.3
**16**	64	79.6 ± 4

EC: Effective concentration is based on at least on three independent experiments. Tested compounds: allantoin (**1**), protocatechuic aldehyde (**5**), malaxinic acid (**9**), rosmarinic acid (**12**), globoidnan A (**14**), comfreyn A (**15**), and caffeic acid ethyl ester (**16**).

## Supplementary Materials

The following are available online at https://www.mdpi.com/1422-0067/21/13/4671/s1. Figure S1: HPLC–UV profiles of the methanol and ethanol extracts at wavelength 280nm. Figure S2: ^1^H NMR Spectrum (600 MHz, CD_3_OD) of compound **15**. Figure S3: ^13^C NMR Spectrum (600 MHz, CD_3_OD) of compound **15**. Figure S4: HSQC Spectrum (CD_3_OD) of compound **15**. Figure S5: HMBC Spectrum (CD_3_OD) of compound **15**. Figure S6: ROESY Spectrum (CD_3_OD) of compound **15**. Figure S7: Globoidnan B (**8**). Figure S8: ^1^H NMR Spectrum (600 MHz, CD_3_OD) of compound **8**. Figure S9: HSQC Spectrum (CD_3_OD) of compound **8**. Figure S10: HMBC Spectrum (CD_3_OD) of compound **8**. Figure S11: Rabdosiin (**11**). Figure S12: ^1^H NMR Spectrum (600 MHz, CD_3_OD) of compound **11**. Figure S13: HSQC Spectrum (CD_3_OD) of compound **11**. **Figure S14**: HMBC Spectrum (CD_3_OD) of compound **11**. **Figure S15**: Globoidnan A (**14**). **Figure S16**: ^1^H NMR Spectrum (600 MHz, CD_3_OD) of compound **14**. **Figure S17**: HSQC Spectrum (CD_3_OD) of compound **14**. **Figure S18**: HMBC Spectrum (CD_3_OD) of compound **14**. Table S1: ^13^C and ^1^H NMR data (*J* in Hz) of compounds **8**, **11**, and **14** (600 MHz, δ ppm, in CD_3_OD).
