# Supplementary material for: LC–ESI–FT–MSn Metabolite Profiling of Symphytum officinale L. Roots Leads to Isolation of Comfreyn A, an Unusual Arylnaphthalene Lignan
Source: Int J Mol Sci. 2020 Jun 30;21(13):4671. doi: 10.3390/ijms21134671 (PMC7370062; doi:10.3390/ijms21134671)
Supplement: Supplementary file 1 [file ijms-21-04671-s001.pdf]

Supplementary Materials

LC- ESI-FT-MS^n metabolite profiling of *Symphytum officinale* L. roots leads to isolation of comfreyn A, an unusual arylnaphthalene lignan

Gilda D'Urso [a,1], Milena Masullo [a,1], Jacqueline Seigner [b], Yvonne M. Holper-Schichl[c], Rainer de Martin [b], Alberto Plaza [d,*], Sonia Piacente [a,*]

[a]*Dipartimento di Farmacia, Università degli Studi di Salerno, Via Giovanni Paolo II, 84084, Salerno, Italy*

[b]*Department of Vascular Biology, Medical University of Vienna, Austria*

[c]*The Procter & Gamble Company Wiedner Gürtel 13, 1100 Vienna, Austria*

[d]*R&D, P&G Health Germany GmbH, Alsfelder Str. 17, 64289 Darmstadt, Germany*

[1]*These authors contributed equally*

List of Supplementary Materials

Figure S1: HPLC-UV profiles of the methanol and ethanol extracts at wavelength 280nm.

Figure S2. ^{1}H NMR Spectrum (600 MHz, CD_3OD) of compound **15**

Figure S3. ^{13}C NMR Spectrum (600 MHz, CD_3OD) of compound **15**

Figure S4. HSQC Spectrum (CD_3OD) of compound **15**

Figure S5. HMBC Spectrum (CD_3OD) of compound **15**

Figure S6. ROESY Spectrum (CD_3OD) of compound **15**

Figure S7. Globoidnan B (**8**)

Figure S8. ^{1}H NMR Spectrum (600 MHz, CD_3OD) of compound **8**

Figure S9. HSQC Spectrum (CD_3OD) of compound **8**

Figure S10. HMBC Spectrum (CD_3OD) of compound **8**

Figure S11. Rabdosiin (**11**)

Figure S12. ^{1}H NMR Spectrum (600 MHz, CD_3OD) of compound **11**

Figure S13. HSQC Spectrum (CD_3OD) of compound **11**

Figure S14. HMBC Spectrum (CD_3OD) of compound **11**

Figure S15. Globoidnan A (**14**)

Figure S16. ^{1}H NMR Spectrum (600 MHz, CD_3OD) of compound **14**

Figure S17. HSQC Spectrum (CD_3OD) of compound **14**

Figure S18. HMBC Spectrum (CD_3OD) of compound **14**

Table S1. ^{13}C and ^{1}H NMR data (*J* in Hz) of compounds **8, 11** and **14** (600 MHz, δ ppm, in CD_3OD)

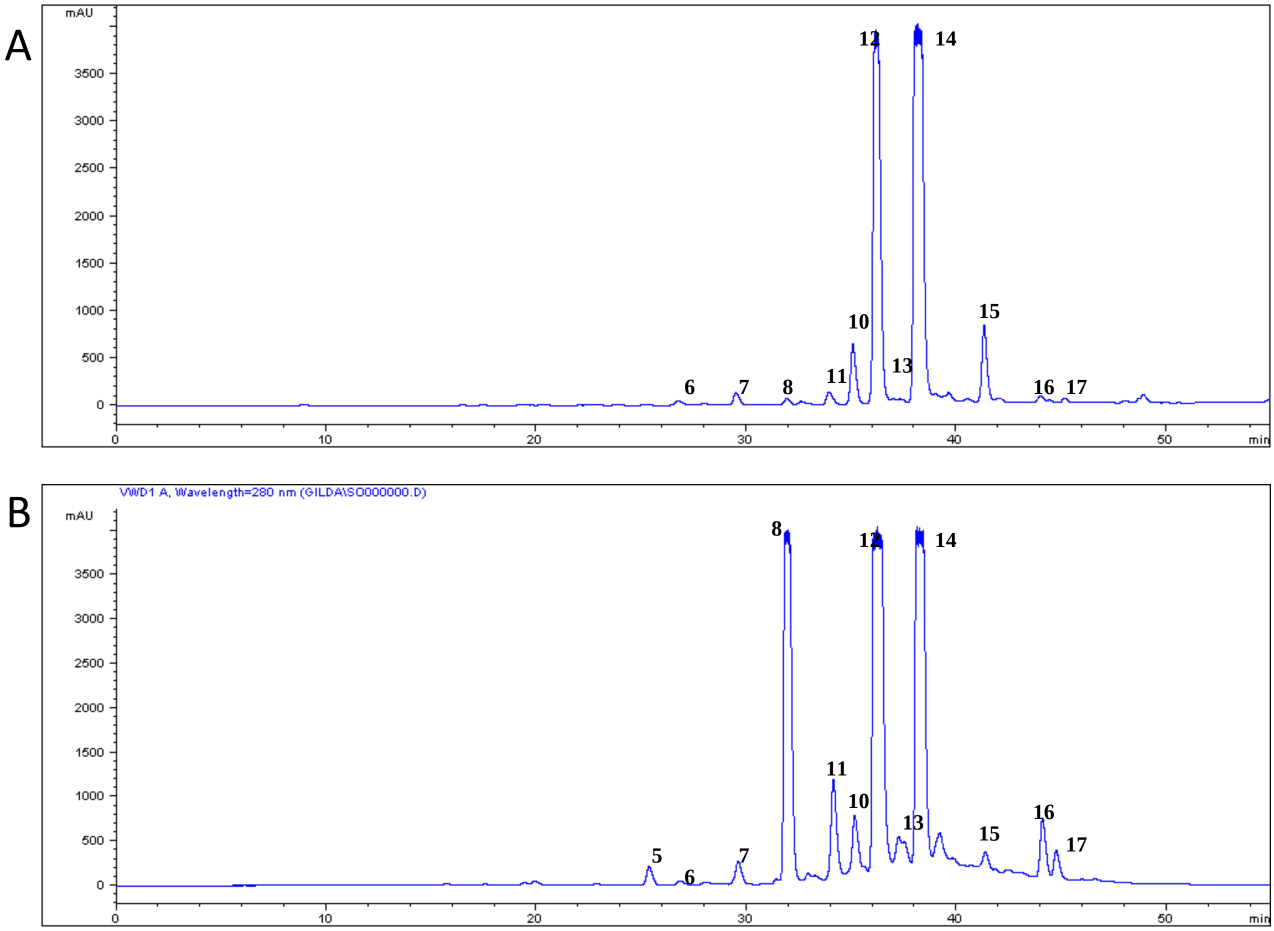

Figure S1: HPLC-UV profiles at wavelength 280 nm: A) methanol extract; B) ethanol extract. Only the highest peaks are numbered according to the corresponding compound identified by LC-ESI-MS and NMR analysis reported in Table 1. The HPLC-UV profiles together with LC-MS analysis confirmed that ethyl esters (**15**, **16** and **17**) are present in the plant and are not artifact.

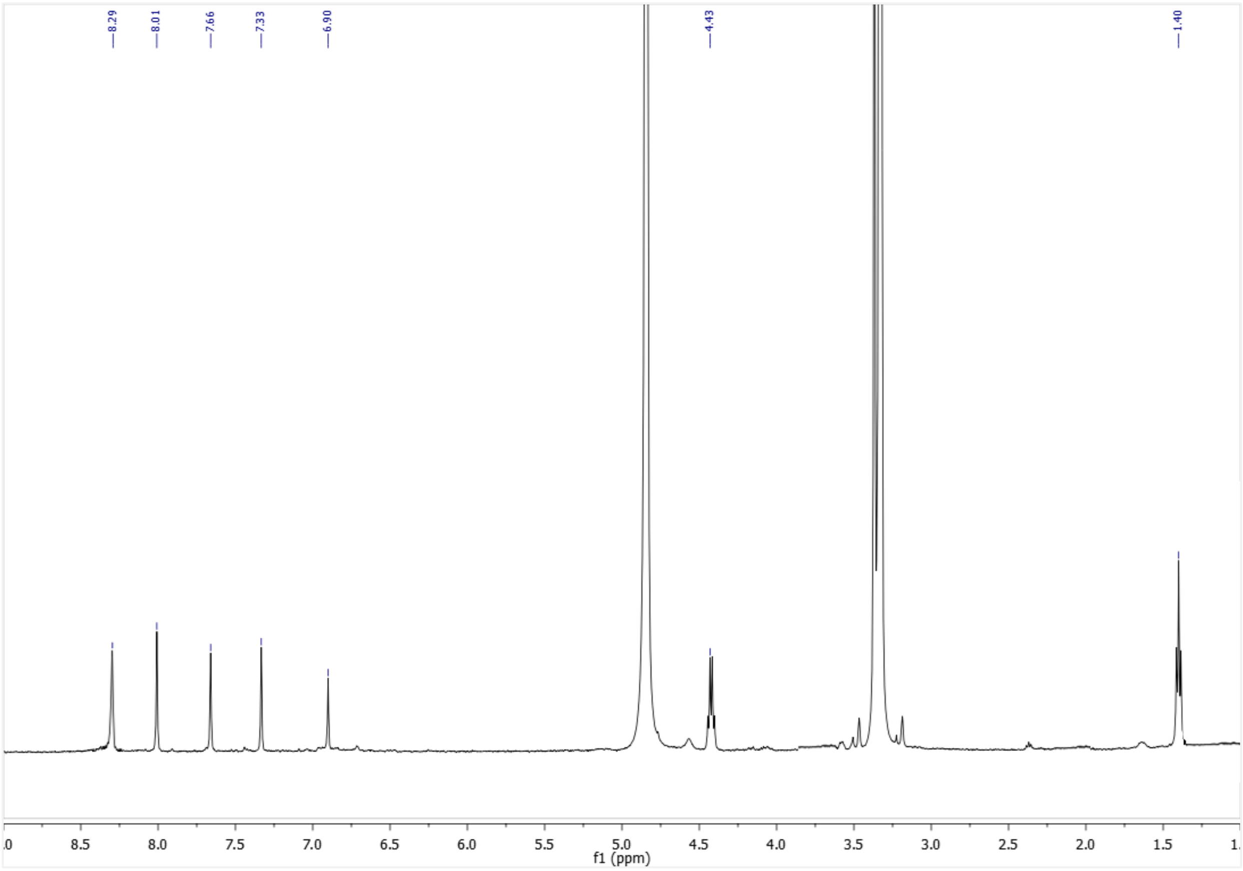

Figure S2. ^{1}H NMR Spectrum (600 MHz, CD_3OD) of compound **15**

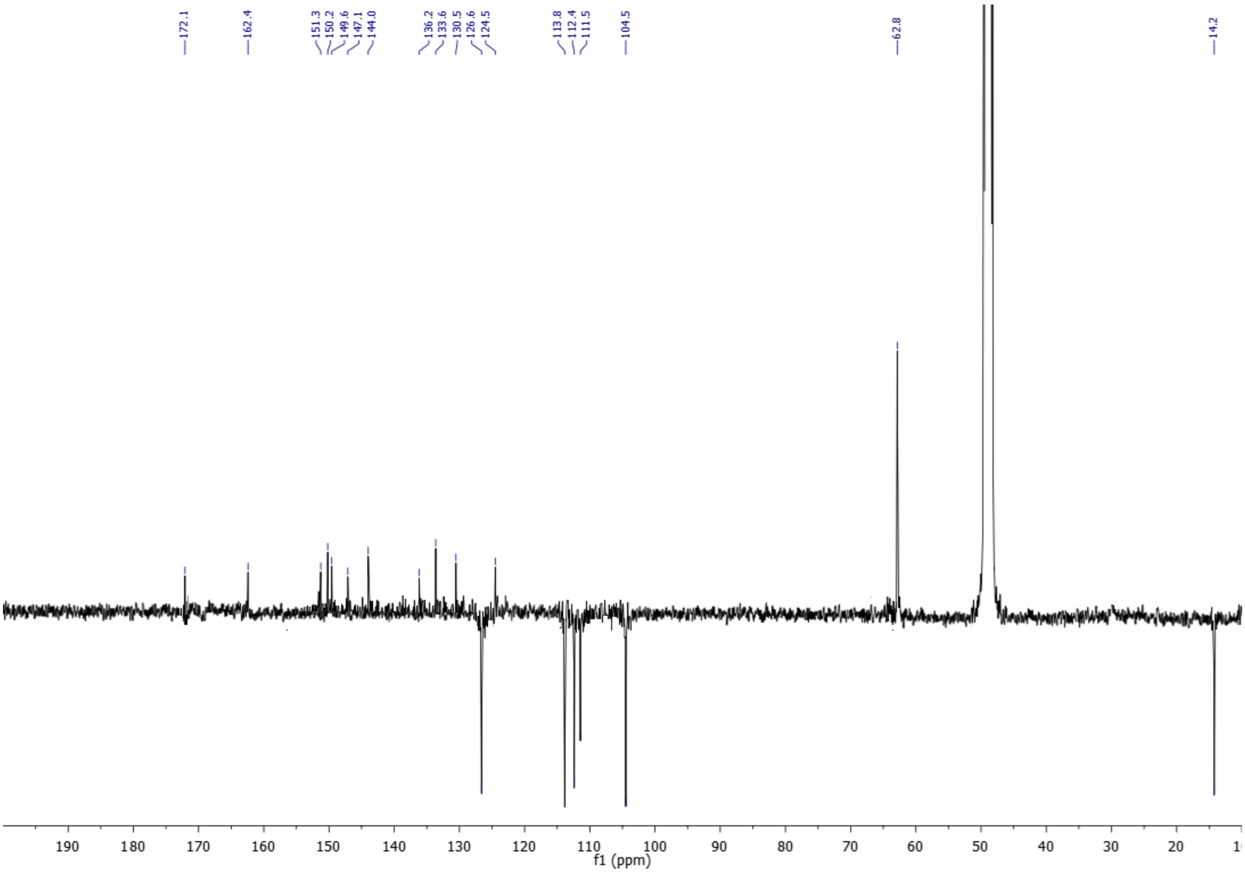

Figure S3. ^{13}C NMR Spectrum (600 MHz, CD_3OD) of compound **15**

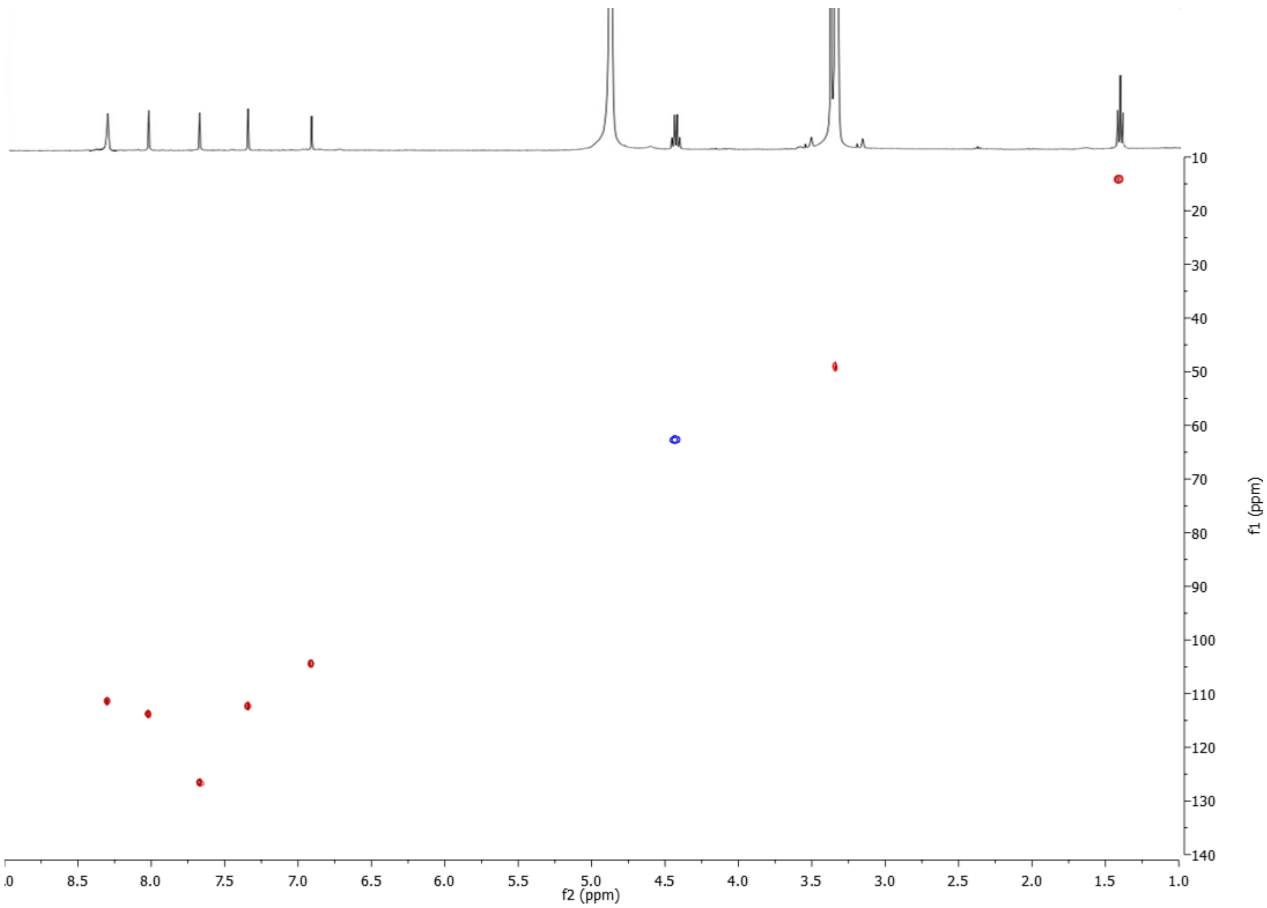

Figure S4. HSQC Spectrum (CD_3OD) of compound **15**

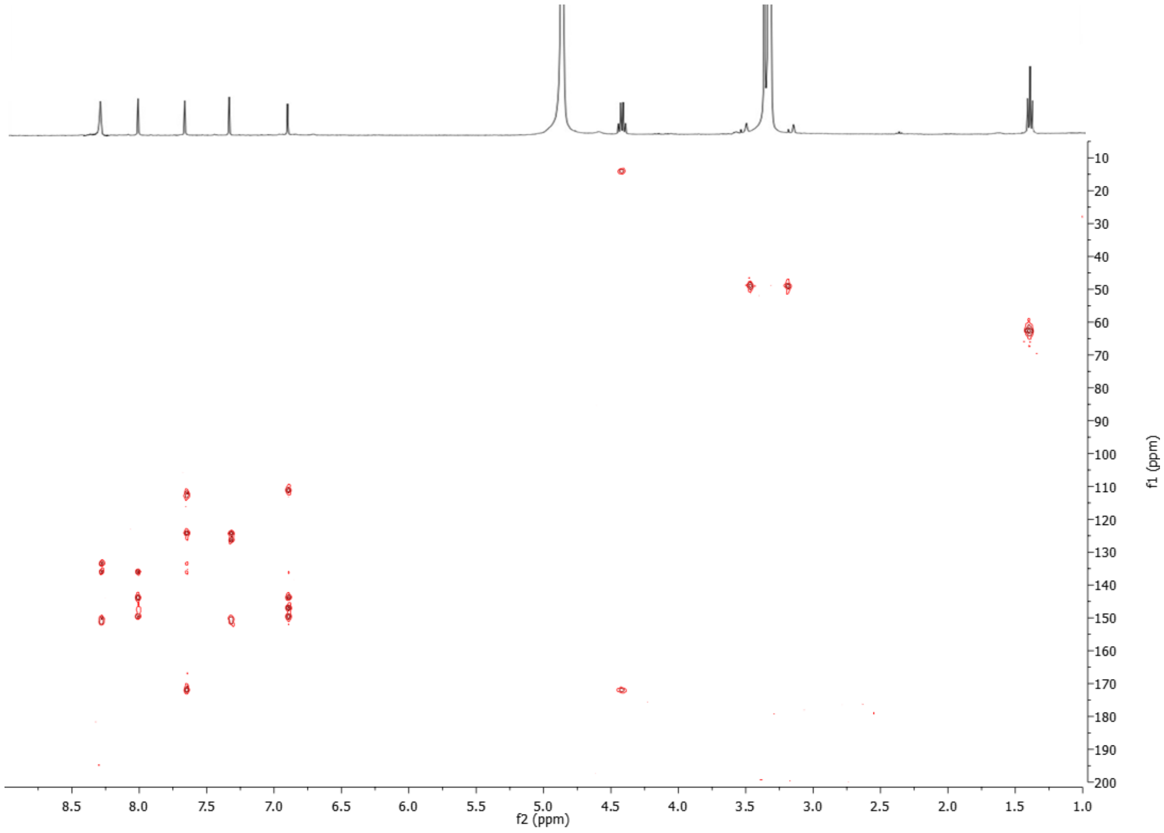

Figure S5. HMBC Spectrum (CD_3OD) of compound **15**

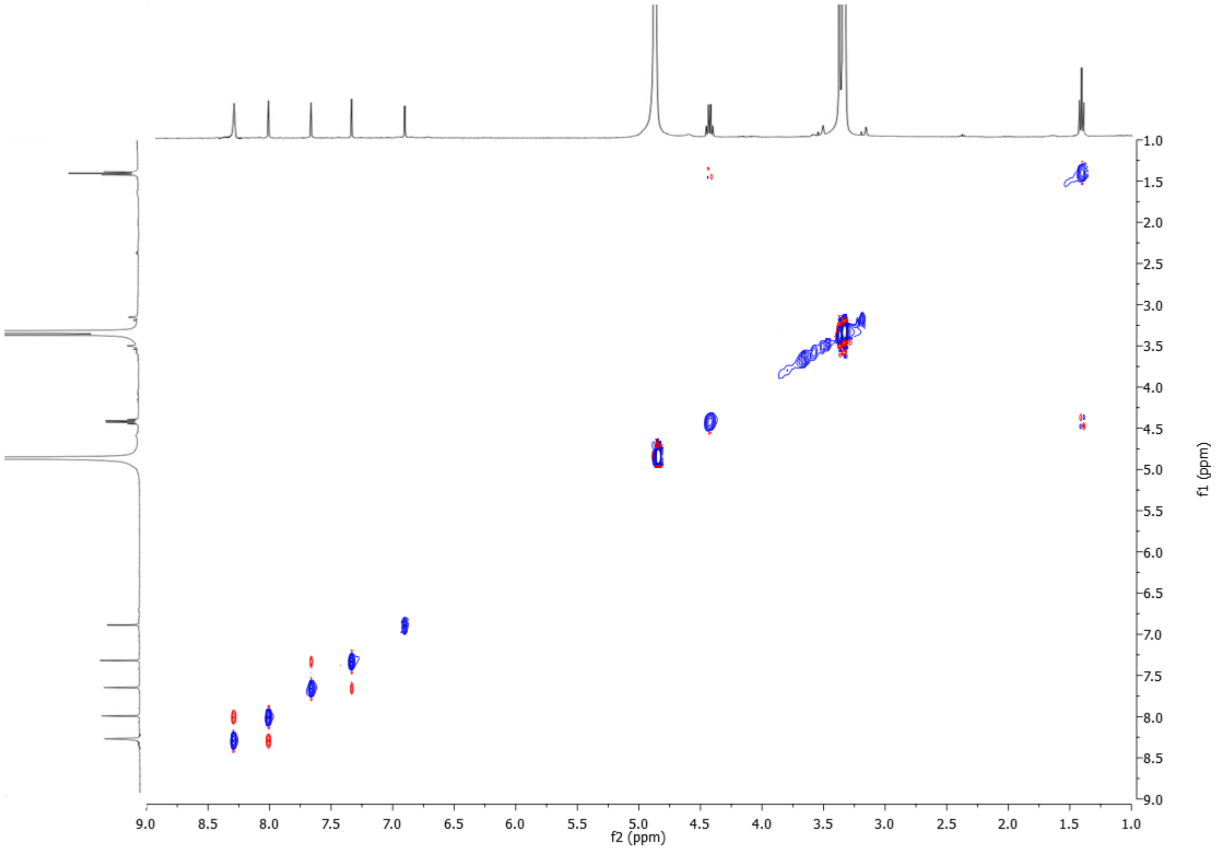

Figure S6. ROESY Spectrum (CD_3OD) of compound **15**

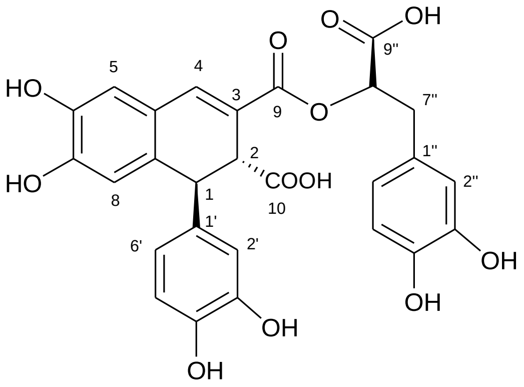

8

Figure S7. Globoidnan B (**8**)

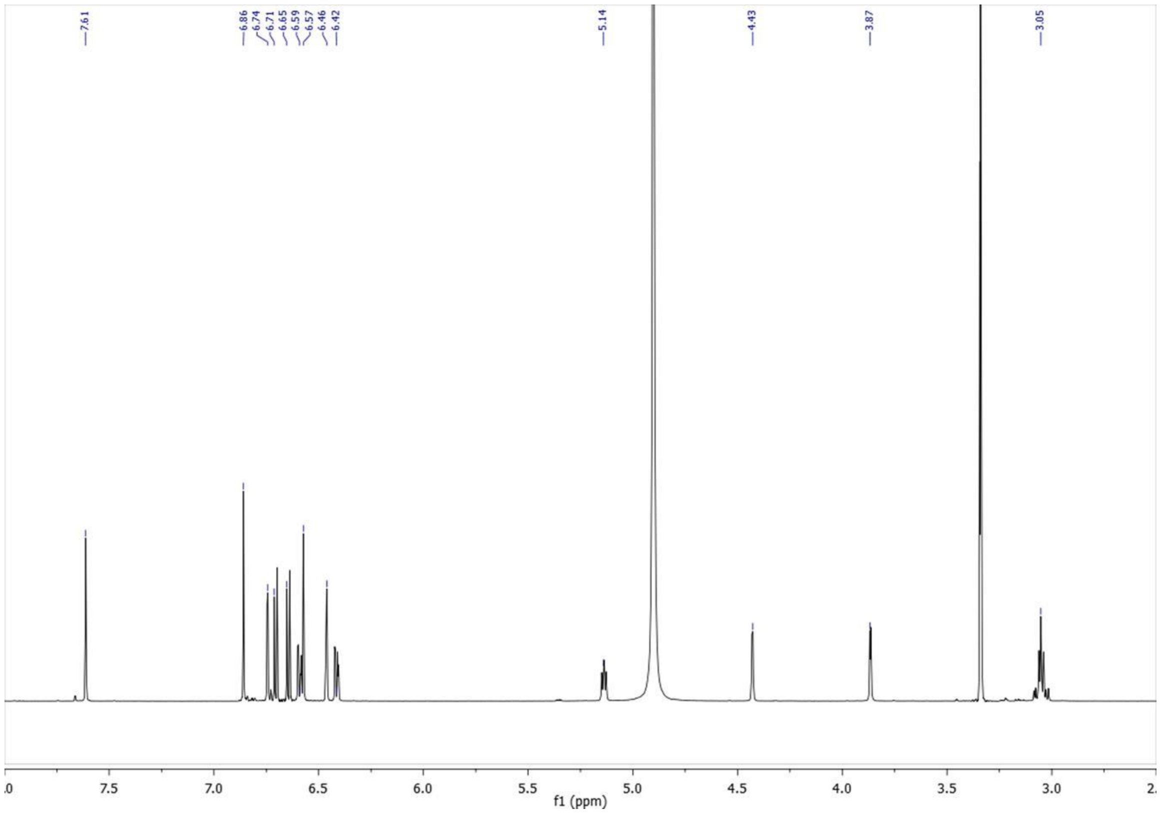

Figure S8. ¹H NMR Spectrum (600 MHz, CD_3OD) of compound **8**

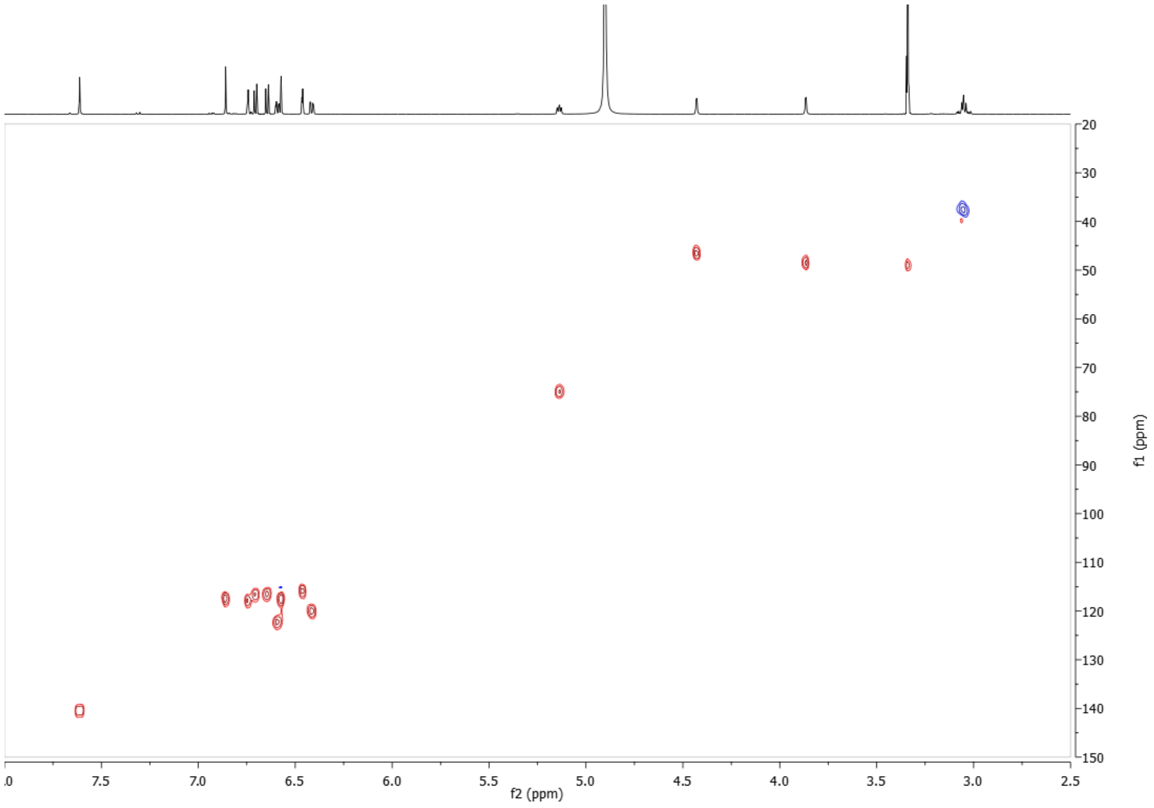

Figure S9. HSQC Spectrum (CD_3OD) of compound **8**

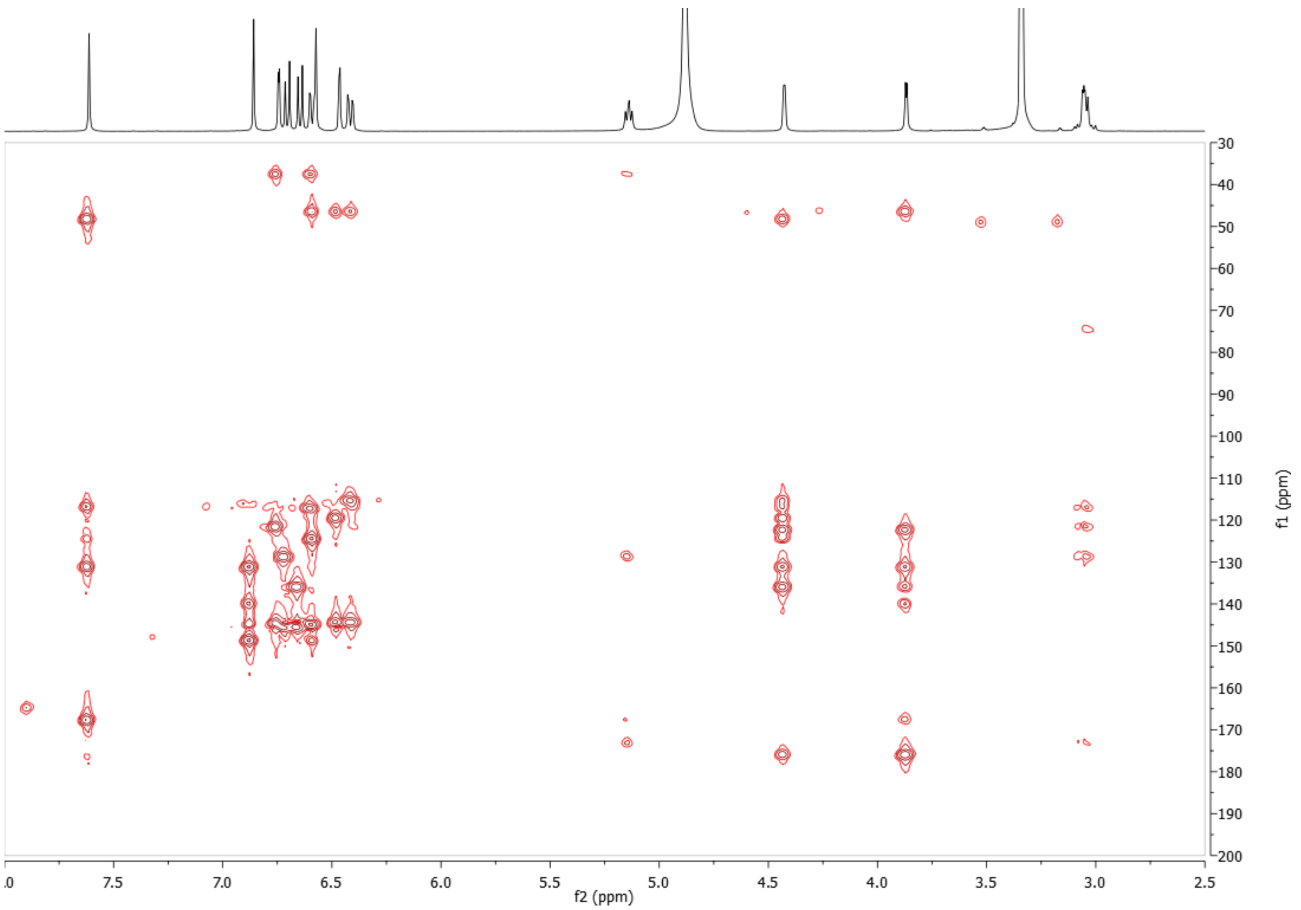

Figure S10. HMBC Spectrum (CD_3OD) of compound **8**

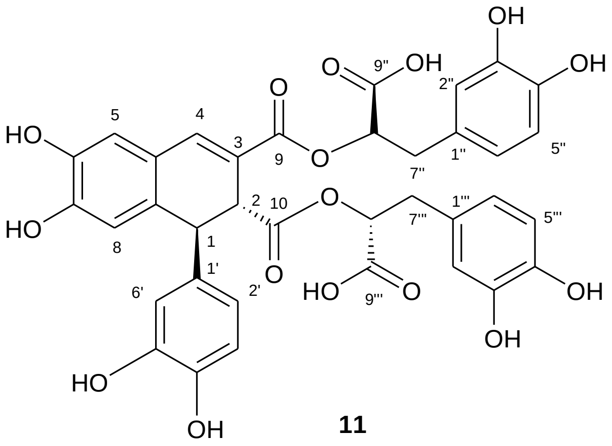

11

Figure S11. Rabdosiin (**11**)

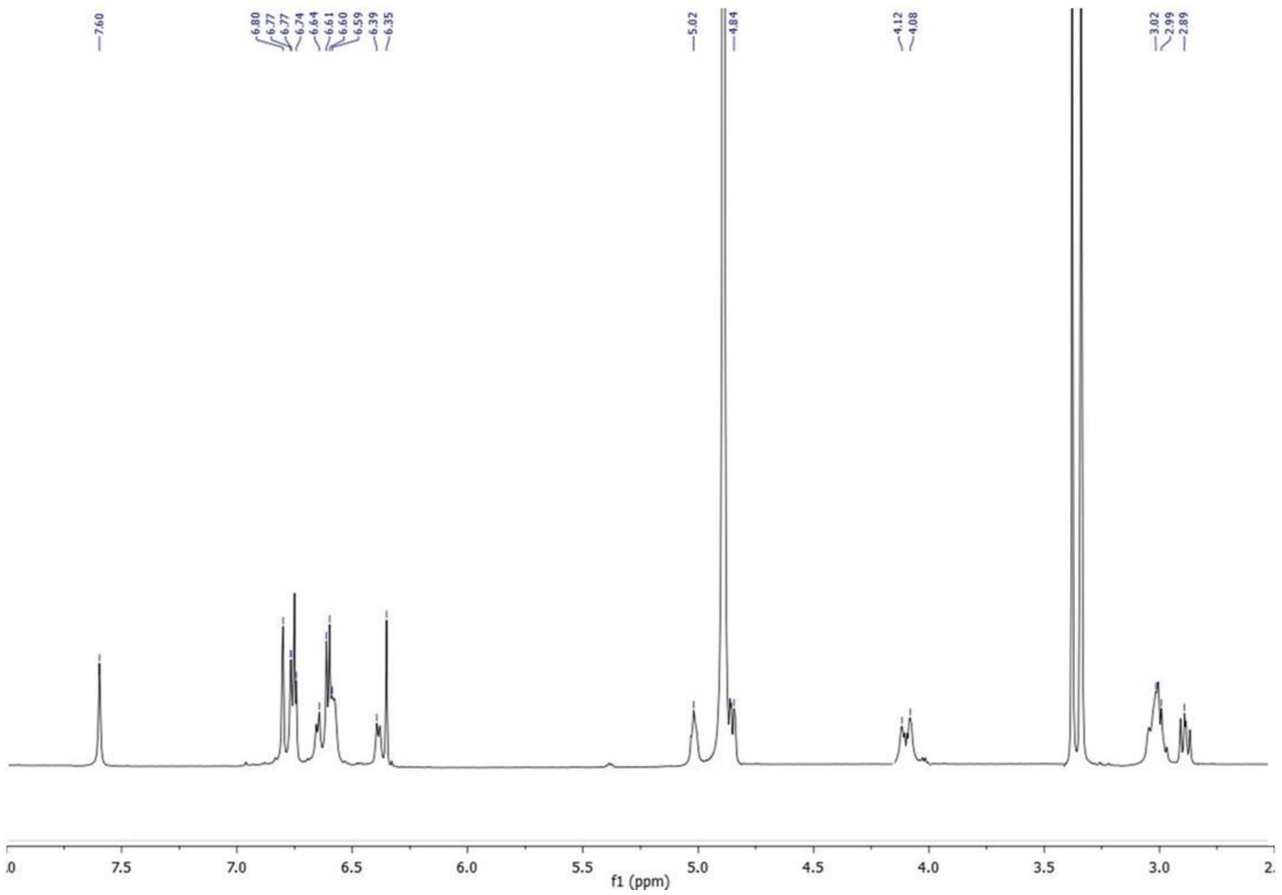

Figure S12. ^{1}H NMR Spectrum (600 MHz, CD_3OD) of compound **11**

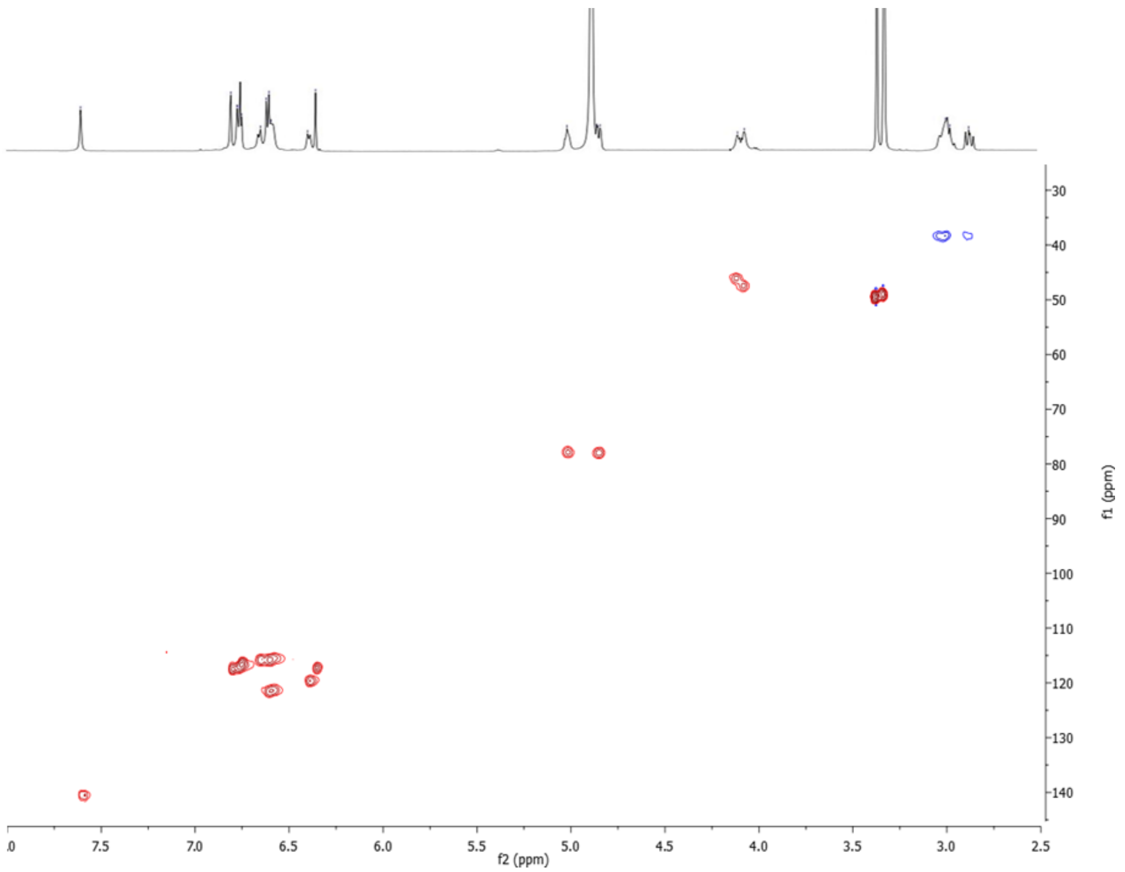

Figure S13. HSQC Spectrum (CD_3OD) of compound **11**

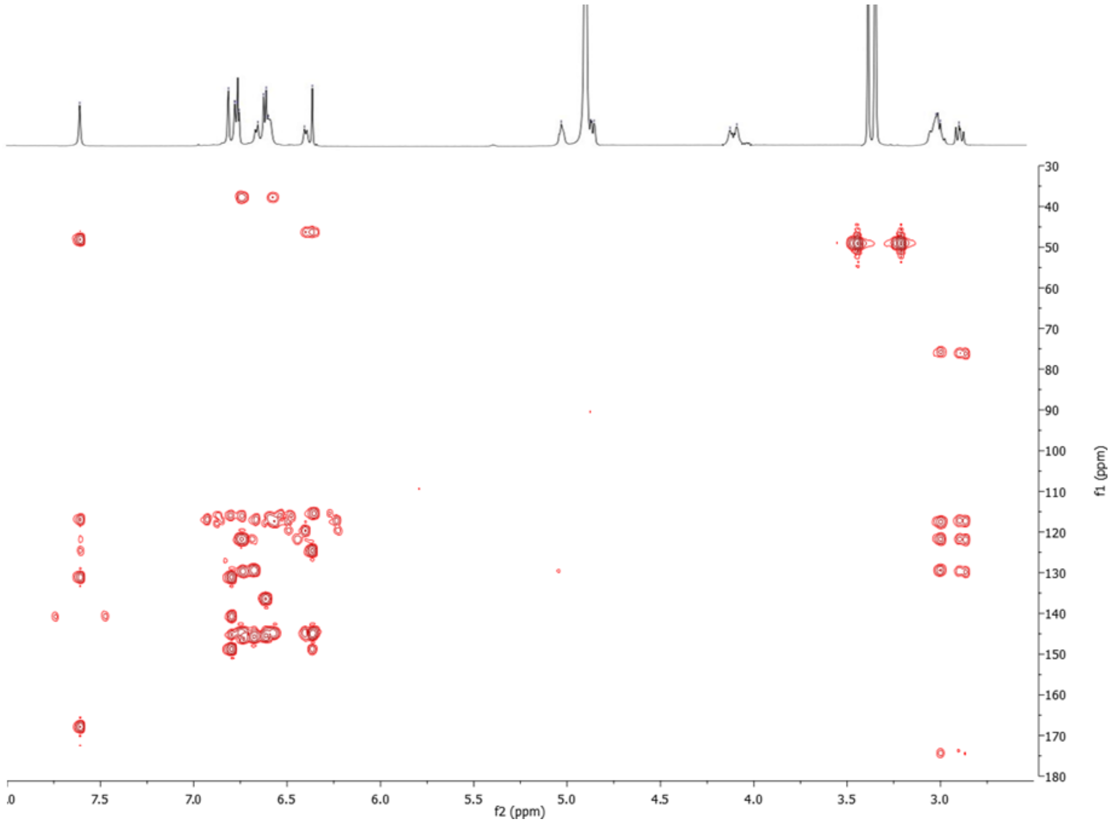

Figure S14. HMBC Spectrum (CD_3OD) of compound **11**

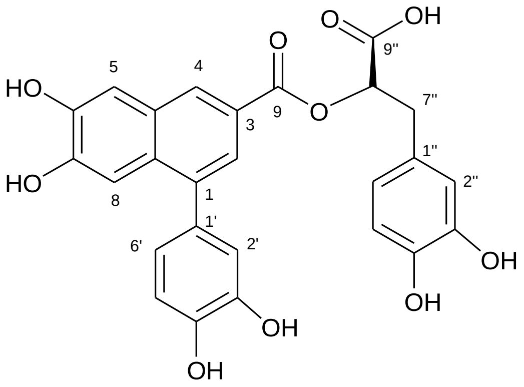

14

Figure S15. Globoidnan A (**14**)

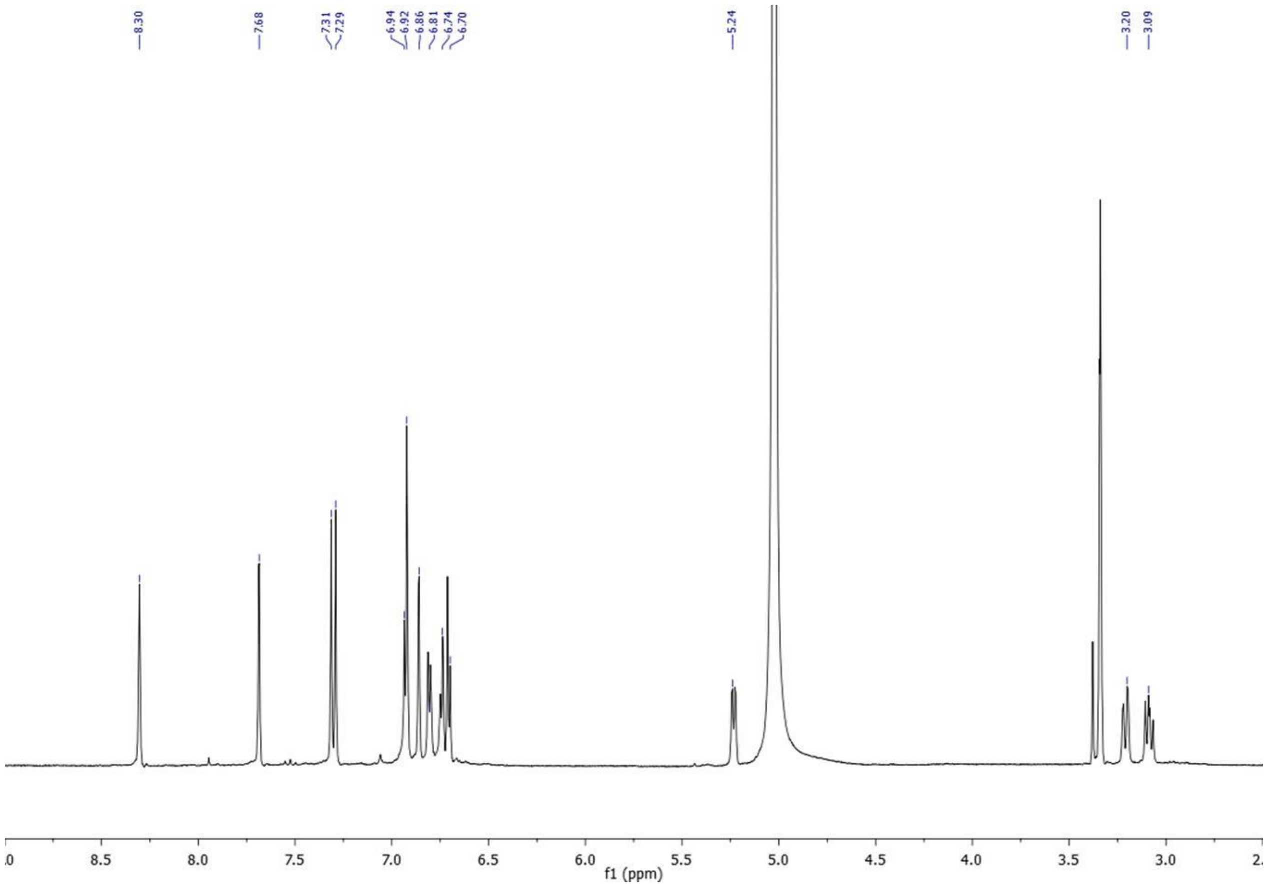

Figure S16. ^{1}H NMR Spectrum (600 MHz, CD_3OD) of compound **14**

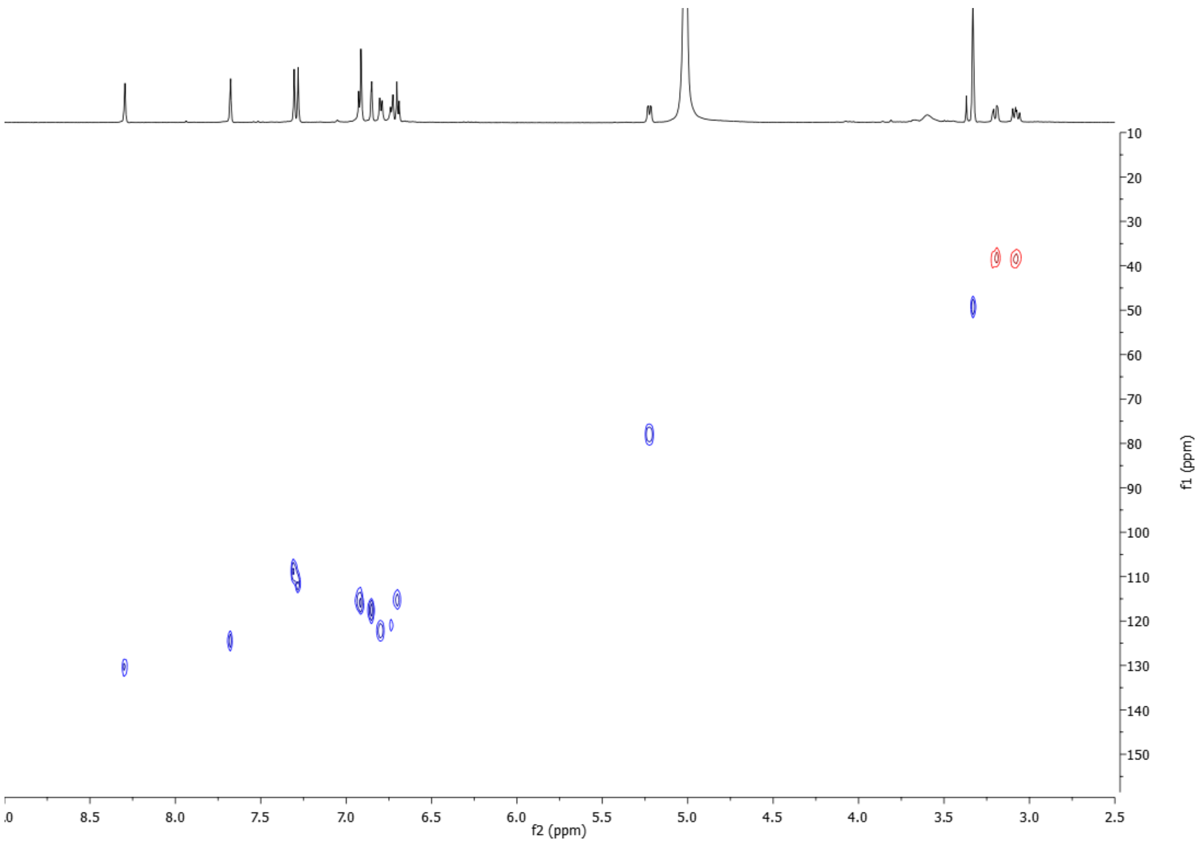

Figure S17. HSQC Spectrum (CD_3OD) of compound **14**

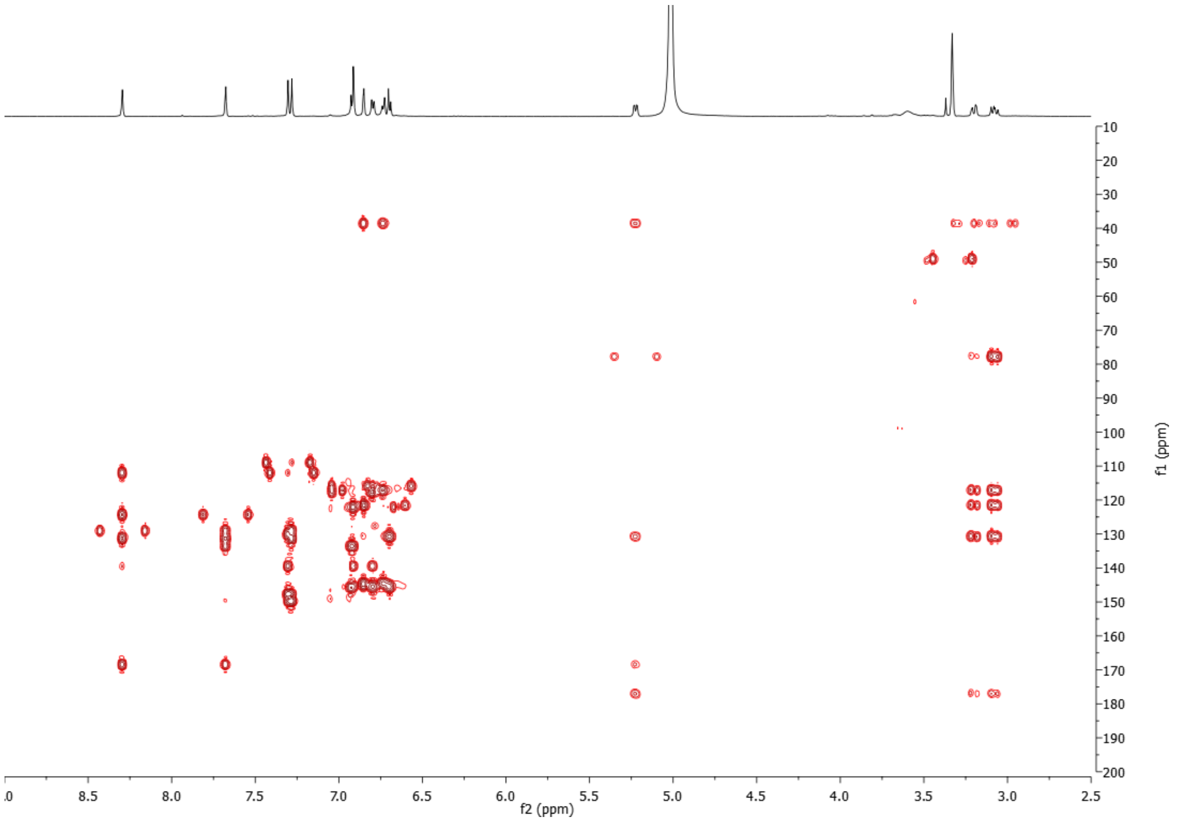

Figure S18. HMBC Spectrum (CD_3OD) of compound **14**

Table S1. ^{13}C and ^{1}H NMR data (*J* in Hz) of compounds **8, 11** and **14** (600 MHz, δ ppm, in CD_3OD)

	**8**		**11**		**14**	
	δ_C	δ_H (*J* in Hz)	δ_C	δ_H (*J* in Hz)	δ_C	δ_H (*J* in Hz)
1	46.4	4.43, d (2.6)	46.1	4.12, d (2.6)	139.4	-
2	48.3	3.87, d (2.6)	47.5	4.08, d (2.6)	124.4	7.68, d (1.6)
3	122.4	-	121.8	-	122.3	-
4	140.6	7.61, s	140.5	7.60, s	130.5	8.30, d (1.6)
4a	124.6	-	124.6	-	129.9	-
5	117.4	6.86, s	117.4	6.80, s	111.4	7.28, s
6	145.1	-	145.1	-	147.6	-
7	148.7	-	148.5	-	149.7	-
8	117.5	6.57, s	117.2	6.35, s	108.6	7.30, s
8a	131.0	-	130.8	-	131.4	-
9	167.9	-	168.1	-	168.5	-
10	175.9	-	173.4	-	-	-
1'	135.9	-	136.2	-	133.5	-
2'	115.8	6.46, d (1.8)	115.8	6.60, d (1.8)	116.1	6.92, d (1.8)
3'	145.5	-	145.2	-	145.4	-
4'	144.5	-	145.5	-	145.5	-
5'	116.5	6.65, d (8.0)	115.8	6.64, d (8.0)	115.3	6.93, d (8.0)
6'	120.0	6.41, dd (8.0, 1.8)	119.6	6.39, dd (8.0, 1.8)	122.1	6.81, dd (8.0, 1.8)
1''	129.0	-	130.8	-	130.7	-
2''	117.7	6.74, d (1.9)	117.1	6.77, d (1.9)	117.5	6.86, d (1.9)
3''	145.7	-	145.7	-	144.5	-
4''	144.7	-	144.8	-	144.7	-
5''	116.5	6.71, d (8.0)	116.3	6.74, d (8.0)	115.5	6.70, d (8.0)
6''	122.2	6.57, dd (8.0, 1.9)	121.4	6.59, dd (8.0, 1.9)	121.0	6.74, dd (8.0, 1.9)
7''	37.6	3.05 (2H), m	38.2	3.02 (2H), m	38.4	3.20, dd (3.5, 14.7)
						3.19, dd (10.3, 14.7)
8''	74.9	5.24, dd (5.0, 7.3)	77.9	5.02, dd (5.0, 7.3)	77.9	5.24, dd (3.5, 10.3)
9''	173.2	-	173.8	-	177.0	-
1'''			130.9	-		
2'''			116.3	6.61, d (1.9)		
3'''			145.5	-		
4'''			145.2	-		
5'''			117.1	6.77, d (8.0)		
6'''			121.4	6.59, dd (8.0, 1.9)		
7'''			38.2	2.99, 2.89, m		
8'''			77.7	4.84, dd (5.0, 7.3)		
9'''			176.1	-		
